# Oxime@Zirconium-Metal–Organic Framework Hybrid Material as a Potential Antidote for Organophosphate Poisoning

**DOI:** 10.1021/acs.inorgchem.3c00121

**Published:** 2023-03-20

**Authors:** Lydia González, Javier D. Martín-Romera, Purificación Sánchez-Sánchez, Jorge A. R. Navarro, Elisa Barea, Carmen R. Maldonado, Francisco J. Carmona

**Affiliations:** Departamento de Química Inorgánica, Universidad de Granada, Avenida Fuentenueva S/N, Granada 18071, Spain

## Abstract

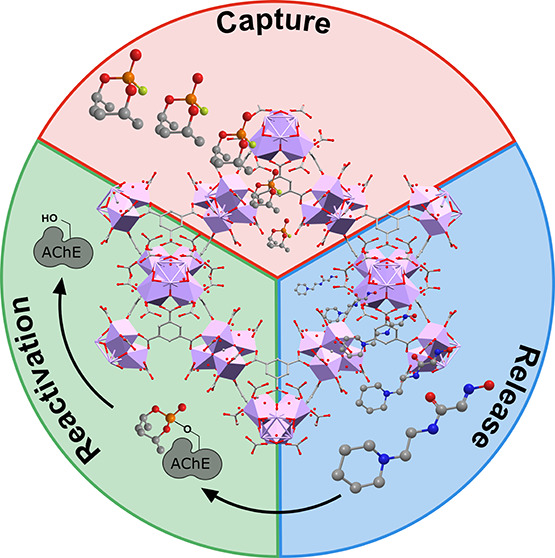

A novel material with dual activity toward organophosphate (OP) poisoning, based on Zr-MOF-808 and neutral oxime RS69N, has been prepared. The hybrid material has a significant drug payload (5.2 ± 0.9 oxime to MOF-808 molar ratio) and shows a sustained oxime release in simulated physiological media, leading to the successful reactivation of OP-inhibited acetylcholinesterase. At the same time, the hybrid system presents an efficient and moderately fast removal rate of a toxic organophosphorus model compound (diisopropylfluorophosphate) from simulated physiological media (*t*_1/2_ = 183 min; 95% removal rate after 24 h).

## Abstract 2

A zirconium-based metal−organic framework as a dual system for the reactivation of organophosphorous inhibited acetylcholinesterase enzyme: capture of toxic diisopropylfluorophosphate and controlled release of RS69N oxime.

Organophosphate (OP) compounds, which include some widely used pesticides and nerve agents, are highly toxic to humans and ecosystems as a consequence of their acetylcholinesterase (AChE) inhibition activity.^1^ Indeed, it is estimated that 110000 deaths per year are globally caused by OP poisoning.^2,3^ It is noteworthy that AChE plays a major role in the correct neurotransmission process by regulating the concentration of neurotransmitter acetylcholine (ACh) in the neuron synapse.^4^ If AChE is not working properly (i.e., due to the strong fixation of OPs to the active serine site), ACh is not hydrolyzed and neuronal ACh receptors become saturated, which results in negative effects on the organism ranging from paralysis to seizures and eventually death.

The coadministration of atropine (to stimulate the heart) and AChE reactivators, generally oximes, is currently the treatment of choice for OP poisoning.^5^ Oximes exert a nucleophilic attack on the P atom of the enzyme–OP adduct, resulting in removal of the phosphoryl moiety from the enzyme active site and the eventual AChE reactivation.^6^ However, while low oxime concentrations lead to inefficient reactivation rates, large amounts of reactivator have been shown to cause inhibition of the enzyme.^6,7^ Thus, oxime dosage is a key parameter during OP-poisoning detoxification. In this sense, the current treatment entails the intravenous administration of a small reactivator dose for multiple times. However, this method generates significant fluctuations in the oxime concentration in the blood, resulting in a poor therapeutic performance.^7^ Therefore, novel systems ensuring a controlled and sustained oxime concentration in the body are highly targeted.^8^ Furthermore, the capability of the reactivator to diffuse toward the central nervous system to reach OP-inhibited brain AChE is another important feature to treat OP poisoning. In this regard, uncharged oxime reactivators are preferred to cationic ones due to the better blood–brain-barrier penetration of the former.^9,10^

On the other hand, metal–organic frameworks (MOFs) are crystalline porous materials characterized by a high surface area, tunable structures, and abundant adsorption sites periodically distributed. These features make MOFs excellent candidates to host bioactive molecules in their cavities (i.e., oximes) and release them in a controlled/gradual manner, acting as drug-delivery systems.^11^ Moreover, for this type of application, carrier toxicity and excretion are aspects to be considered. Recently, these issues have been extensively revised.^12^ Concerning OP poisoning, only a few investigations have reported the use of MOFs as carriers of oximes. For example, a titanium aminoterephthalate MOF (MIL-125-NH_2_) demonstrated to efficiently adsorb and rapidly release the prototypical antidote 2-[(hydroxyimino)methyl]-1-methyl-pyridinium chloride (pralidoxime).^13^ In another recent study, the iron-based MOF MIL-88B was tested for the long-term and continuous delivery of the same oxime, which was effective in the treatment of intragastric-poisoned mice with sarin.^7^ Despite these interesting results, in these studies the exceptional adsorption capacity of MOFs has not been fully exploited. In this sense, MOFs could remove the toxic OP compounds from body fluids, keeping them trapped inside their porous matrixes, and simultaneously host and release oximes in a controlled manner. In particular, MOFs based on Zr_6_O_4_(OH)_4_ secondary building units have proven to have an elevated adsorption capacity of OPs due to the Pearson hard-acidic nature of Zr(IV) sites and a concomitantly strong affinity for phosphate compounds. Thus, the combination of both capabilities, namely, the capture of toxic OP compounds and the sustained release of noncharged AChE reactivators, could lead to improved treatments against OP poisoning.^14,15^ In this regard, our research group has recently demonstrated the dual behavior of zirconium metal–organic polyhedra (Zr-MOP) for the capture of diisopropylfluorophosphate and the continuous delivery of pralidoxime.^16^ However, as far as we know, this dual functionality has not been explored yet on extended MOFs.

On the basis of the above considerations, in this work, we have prepared a novel oxime@zirconium-based MOF (Zr-MOF) material to treat OP poisoning. In particular, we have selected the 6-connected Zr-MOF [Zr_6_O_4_(OH)_4_(trimesate)_2_(formate)_6_] (MOF-808), with high accessibility to its adsorption Zr(IV) sites and mesopores to host bulky guest molecules, as the carrier of the noncharged AChE reactivator oxime 2-(hydroxyimino)-*N*-2-(piperidin-1-yl)ethylacetamide (RS69N). The controlled release of this molecule allows the successful reactivation of phosphorylated AChE enzyme. Additionally, we have evaluated the ability of this hybrid material to remove the toxic diisopropylfluorophosphate (DIFP) in simulated physiological media (Scheme 1).

**Scheme 1:**
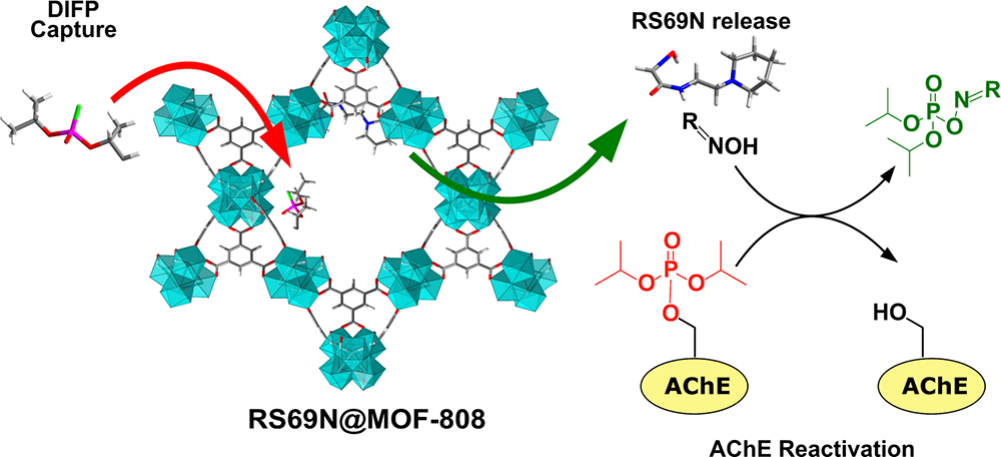
Schematic Representation of Dual DIFP Capture and RS69N Release by RS69N@MOF-808 Material The released RS69N oxime is capable of reactivating previously inhibited AChE enzyme.

The suitability of MOF-808 as a carrier of the RS69N drug was evaluated by means of Monte Carlo Metropolis computational modeling using Adsorption Locator Module of Biovia *Materials Studio*.^17^ The model confirms a high oxime loading capacity of up to 52 RS69N molecules per crystal cell, which are accommodated in MOF-808 mesopores. It is noteworthy that the oxime-loaded MOF structure still has a 7.36% accessible volume, to a 1.4 A radii probe molecule, and it is able to accommodate five DIFP molecules (Figure S1). Both MOF-808 and RS69N oxime were prepared according to previously reported methods.^9,18^ It is noteworthy that nanometric MOF-808 was synthesized following an environmentally friendly method previously described by us.^18^ Once these materials were fully characterized (see the Supporting Information, SI), we proceeded to encapsulate the AChE reactivator into the porous matrix by means of a solid–liquid impregnation strategy.

In a typical experiment, MOF-808 was suspended in a methanolic solution of RS69N, and the mixture was stirred until complete evaporation of the solvent in order to force the incorporation of oxime into the pores (see the SI). The resulting solid, RS69N@MOF-808, was thoroughly washed with water, lyophilized, and kept in a refrigerator. IR spectroscopy first confirmed the successful loading of RS69N into the cavities of the Zr-MOF because some of the characteristic bands of oxime were present in the hybrid material (Figure 1a). Moreover, powder X-ray diffractograms revealed that RS69N@MOF-808 maintains the structural integrity of pristine MOF-808 (Figure S2). Scanning electron microscopy (SEM) images showed that the hybrid material is formed by MOF-808 particles with a homogeneous size of 485 ± 35 nm (Figure 1b), which are expected to be excreted via the liver and spleen and, therefore, to be less harmful than smaller particles (15–200 nm).^12^ In order to quantify the drug loading, the hybrid material was digested in a NaOD solution (10 M) and the supernatant was analyzed by NMR, allowing us to estimate a 5.2 ± 0.9 oxime-to-MOF-808 molar ratio (Figure S3).

**Figure 1:**
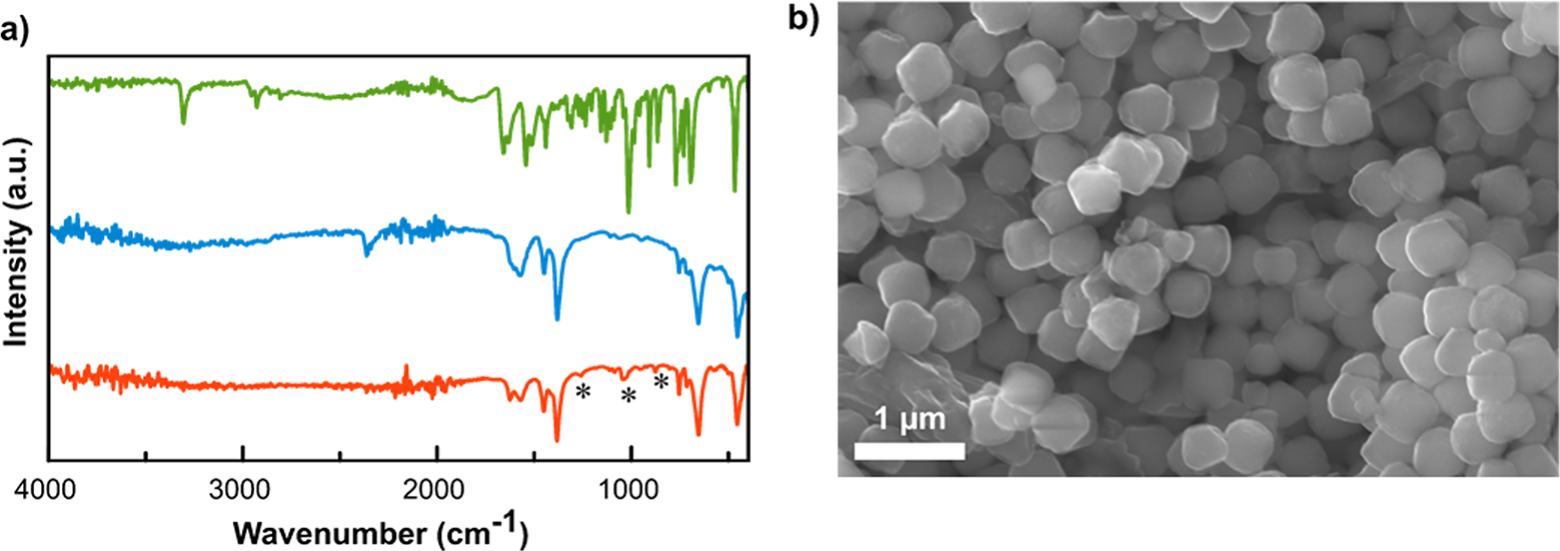
(a) IR spectra of RS69N (green), MOF-808 (blue), and hybrid RS69N@MOF-808 (red). The characteristic bands of RS69N are highlighted with asterisks. (b) SEM image of RS69N@MOF-808. Scale bar: 1 μm.

Aiming at testing the potential of RS69N@MOF-808 to reactivate AChE, we first investigated the release of RS69N in aqueous media [100 mM phosphate-buffered saline (PBS), 37 °C] by NMR spectroscopy using dimethylacetamide (DMA) as the internal reference (Figures S4 and S5 and Table S1). The results revealed a first burst effect (5.8% of cumulative release) followed by a gradual release of the loaded drug (9.9% of cumulative release after 180 min and 37.5% after 24 h; Figure 2). It is noteworthy that our system allows a continuous dosage of RS69N oxime over time, which seems to be optimal to achieve an efficient treatment of OP poisoning with AChE reactivators.^8^

**Figure 2:**
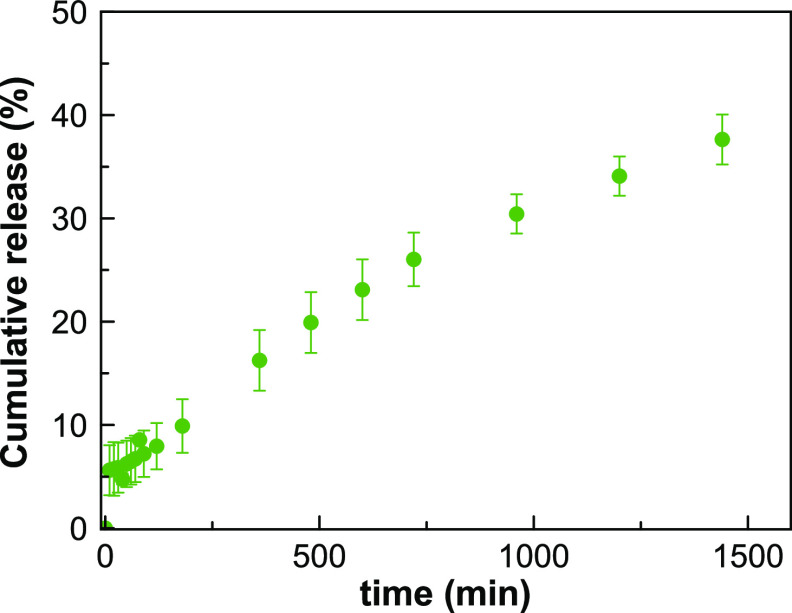
Cumulative release profile of RS69N drug from a suspension of RS69N@MOF-808 in simulated physiological media. Experimental conditions: 20 mg of RS69N@MOF-808, 0.5 mL of PBS (100 mM, pH = 7.4), room temperature.

Then, the ability of the released oxime in the reactivation of AChE was assayed. First, we confirmed in which concentration range free RS69N successfully reactivates AChE activity (97 ± 3% and 25 ± 5% of AChE reactivation for oxime concentrations of 5 × 10^–3^ and 5 × 10^–4^ M, respectively). Afterward, 70.4 ± 1.4% inhibited AChE was exposed to the supernatants obtained from RS69N@MOF-808 incubation in a PBS buffer ([RS69N] = 3.2 × 10^–3^ M), leading to a full AChE reactivation (100% ± 8%; Table S2). These results suggest that MOF-808 does not negatively affect the ability of the released oxime to reactivate AChE.

As mentioned above, Zr-MOFs, such as MOF-808,^19^ have demonstrated a good performance in the removal of OP compounds in water. This prompts us to evaluate the ability of RS69N@MOF-808 to decontaminate DIFP solutions in simulated physiological media (100 mM PBS, 37 °C). In our studies, a suspension of the hybrid material (20 mg) in an aqueous DIFP solution (2.9 × 10^–2^ M, 500 μL) was stirred at room temperature for 24 h and the nerve agent simulant concentration was monitored by gas chromatography, using DMA as the internal reference. Interestingly, DIFP was quickly captured from the solution, reaching 30 and 95% removal after 90 and 1440 min, respectively. The kinetics were successfully fitted to a pseudo-second-order adsorption model (*k* = 5. 10^–3^ mol_MOF_·mol_DIFP_^–1^·min^–1^ and *t*_1/2_ = 183 min; Figures 3 and S6). A control experiment was also carried out to evaluate the ability of pristine MOF-808, with empty pores, to remove DIFP from a PBS solution. As is shown in Figure 3, MOF-808 shows slightly faster DIFP adsorption kinetics than the RS69N@MOF-808 system (*k* = 9 × 10^–3^ mol_MOF_·mol_DIFP_^–1^·min^–1^ and *t*_1/2_ = 99 min; Figure S7). Therefore, these results indicate that the encapsulation of oxime inside the cavities of MOF-808 does not have a significant effect on the ability of the hybrid material to remove this toxic OP compound. Moreover, it should be highlighted that relatively large particles (>200 nm), such as RS69N@MOF-808, are excreted via the liver and spleen, ensuring the successful removal of DIFP from the body.^12^

**Figure 3:**
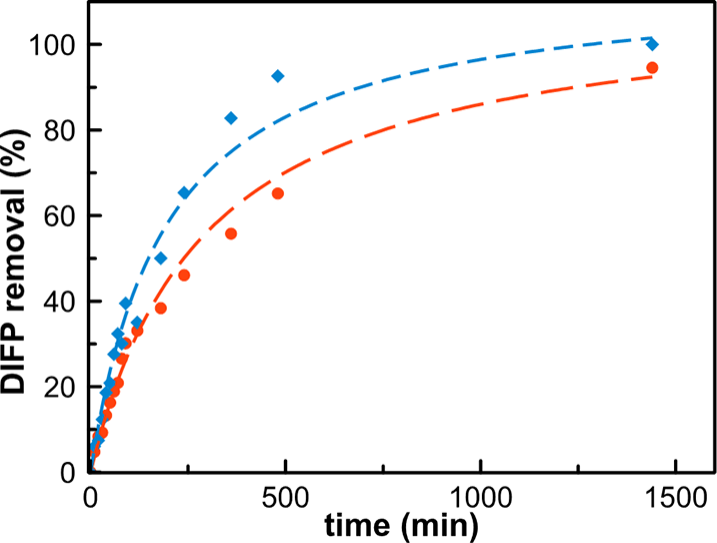
Cumulative removal of DIFP in simulated physiological media by MOF-808 (blue curve) and the RS69N@MOF-808 hybrid material (red curve). Experimental conditions: 20 mg of porous material, 0.5 mL of PBS (100 mM, pH = 7.4), room temperature. Internal reference: DMA.

In order to elucidate the nature of the DIFP decontamination process (adsorption vs catalytical degradation), additional ^31^P and ^1^H NMR studies using DMA as the internal reference were performed. In a typical experiment, RS69N@MOF-808 (20 mg) was suspended in an equimolar mixture of DIFP and DMA (2.9 × 10^–2^ M) in simulated physiological media (100 mM PBS, 37 °C). After 24 h, the supernatant was separated from the solid by centrifugation and analyzed by NMR. ^31^P NMR of the supernatant demonstrated the presence of two P species, namely, diisopropylhydrogen phosphate (DIFP degradation product) and pristine DIFP (Figure S8). Moreover, ^1^H NMR allowed us to quantify the extent of the degradation process, confirming that approximately 8.7% of the original DIFP was hydrolyzed (Figure S9). In addition, the solid was treated with deuterated dimethyl sulfoxide, and the extracted solution was also analyzed by ^31^P NMR, confirming that only DIFP (not its degradation product) was adsorbed into the porous matrix (Figure S10). Thus, NMR assays, together with chromatographic studies, reveal that most of the toxic DIFP is adsorbed into the porous matrix (74.2%), while 8.7% of its degradation product and only 17.2% of free DIFP remained in the solution after 24 h.

Finally, we performed an additional enzymatic assay to test the detoxifying effect of hybrid RS69N@MOF-808 in the presence of DIFP. Specifically, 20 mg of RS69N@MOF-808 was dispersed in a DIFP solution (2.9 × 10^–2^ M, 0.5 mL) in PBS (100 mM), and the mixture was kept at 37 °C for 24 h. A DIFP solution (2.9 × 10^–2^ M, 0.5 mL) in PBS (100 mM) was also prepared as a negative control. After 24 h of incubation, the inhibition effect of the supernatants on the AChE enzymatic activity was evaluated (see the SI). While the control DIFP solution inhibited the AChE activity by 63.5 ± 1.3%, the supernatant collected from the RS69N@MOF-808 and DIPF mixture showed an inhibition activity of only 13.7 ± 1.5% (Table S2). Thus, the removal of this toxic compound by the hybrid material significantly reduces (78.4%) the inhibitory effect of DIFP on the AChE enzyme.

In conclusion, the exceptional porosity and high density of the OP sorption sites of MOF-808 have been exploited to prepare a novel hybrid material RS69N@MOF-808 showing dual behavior toward the treatment of OP poisoning. Specifically, the material is able to host and gradually release the neutral oxime RS69N and fully reactivate DIFP-inhibited AChE. At the same time, RS69N@MOF-808 is also capable of capturing DIFP from a PBS solution, preventing interaction of the organophosphorus compound with AChE and thereby remarkably reducing its toxic effect.

## Supporting Information Available

Details of experimental procedures, synthesis of compounds, powder X-ray diffractograms, ^1^H and ^31^P NMR spectra, kinetic fittings, computational modeling, UV–vis absorption data, and thermogravimetric analysis, including Figures S1–S11 and Tables S1 and S2 (PDF)
