# Supplementary material for: Oxime@Zirconium-Metal–Organic Framework Hybrid Material as a Potential Antidote for Organophosphate Poisoning
Source: Inorg Chem. 2023 Mar 20;62(13):5049–53. doi: 10.1021/acs.inorgchem.3c00121 (PMC10074384; doi:10.1021/acs.inorgchem.3c00121)
Supplement: Supplementary file 1 — ic3c00121_si_001.pdf [file ic3c00121_si_001.pdf]

SUPPORTING INFORMATION

Oxime@Zirconium-Metal-organic Framework Hybrid Material as Potential Antidote for Organophosphate Poisoning

Lydia González, [a,‡] Javier D. Martin-Romera, [a,‡] Purificación Sánchez-Sánchez,[a] Jorge A. R. Navarro,[a] Elisa Barea,[a] Carmen R. Maldonado,*[,a] Francisco J. Carmona *[,a]

[a] Departamento de Química Inorgánica, Universidad de Granada, Av. Fuentenueva S/N, 18071 Granada, Spain.

Corresponding Authors:

Carmen R. Maldonado:crmaldonado@ugr.es, Francisco J. Carmona: fjcarmona@ugr.es

Table of content

EXPERIMENTAL SECTION 2

S.1. General Methods of characterization 2

S.2. Synthesis 2

S.2.1. Synthesis MOF-808 2

S.2.2. Synthesis RS69N 3

S.2.3. RS69N encapsulation into MOF-808 3

S.3. RS69N release from RS69N@MOF-808 4

S.4. Enzymatic assays 4

S.5. DIFP removal studies 5

S.5.1. Gas chromatography studies 5

S.5.2. ^{1}H and ^{31}P NMR studies 5

S.6. Computational modelling (Adsorbate locator) 5

FIGURES 7

BIBLIOGRAPHY 18

EXPERIMENTAL SECTION

S.1. General Methods of characterization

All chemicals were commercially available at commercial sources and used without further purification. **MOF-808** was synthesized using an Anton Paar Monowave 300 reactor. Powder X-ray diffraction data (PXRD) were obtained at room temperature on a D2 PHASER Bruker diffractometer using Cu Kα radiation (λ = 1.5418 Å) and collected in the 5°-35° 2θ range, with steps of 0.02° and time per step of 1.0 sec. Prior to each measurement, the samples were manually grounded in an agate mortar and then deposited in the hollow of a zero-background silicon sample holder. Infrared spectra were collected in a Fourier transform infrared spectrophotometer Bruker Tensor 27 (32 scans, resolution = 2 cm^{-1}). ^{1}H and ^{31}P Nuclear Magnetic Resonance Spectroscopy data were obtained in a BRUKER Nanobay Avance III HD High Definition 400 MHz (2-channel) NMR spectrometer. Thermogravimetric Analysis (TGA) were carried out by a Thermogravimetric analyser METTLER-TOLEDO mod. TGA STAR system under air flow (20 mL min^{-1}) running from room temperature to 950 °C with a heating rate of 10 °C min^{-1} (CIC, University of Granada). Scanning electron microscopy (SEM) images were obtained on a Zeiss SUPRA40VP (FESEM) system. DIFP adsorption experiments were carried out in an Agilent 8860 GC Chromatograph. This chromatograph has FID detector and a 16 port autosampler. An HP-5 column of 50 m length, 0.320 mm diameter and 1.05 um thickness was used, which allows working from -60 °C to 325 °C. Enzymatic assays were performed in a Tecan Infinite® 200 PRO NanoQuant.

S.2. Synthesis

S.2.1. Synthesis MOF-808

MOF-808 was synthesized according to a previously published method.[1] $ZrOCl_2 \cdot 8H_2O$ (1.288 g, 4.00 mmol) and trimesic acid (0.280 g, 1.33 mmol) were added to a mixture of formic acid / H_2O (10 mL / 10 mL) in a 30 mL reaction vial and sonicated for 5 min. Afterwards, the suspension was heated up to 95 °C (heating ramp of 60 min), then kept at 95 °C for 1 hour and finally, a fast cooling-step of 2 min to reach room temperature. The white powder was centrifuged (2,486 *g* / 5 min) and washed three times with H_2O and once with acetone. To remove the solvent trapped inside the pores, the material was

activated under dynamic vacuum at 100 ºC during 8 h. TGA residue (calc./exp.): 54.21/54.61 % (**Figure S11**). Yield: 71.8 %

S.2.2. Synthesis RS69N

The synthesis of the oxime was carried out following the procedure previously described in the literature.[2,3] Hydroxylamine hydrochloride (0.69 g, 10 mmol), 50% ethyl glyoxylate in toluene (1.98 mL, 10 mmol), acetonitrile (7.3 mL) and H_2O (0.8 mL) were added in a 25 mL round-bottom flask and stirred for 5 min at room temperature. Triethylamine (1.40 mL, 10 mmol) was then added dropwise and the mixture was stirred for 1 h. Then, the solution was concentrated via rotary evaporation to give a white residue. The solid was dissolved in a mixture of H_2O / diethyl ether (15 mL / 15 mL), the organic layer separated and the remaining aqueous layer extracted twice with diethyl ether (20 mL). The resulting organic layers were combined and washed with brine, dried over anhydrous magnesium sulphate and concentrated under rotary evaporation. The obtained syrup, ethyl glyoxylate oxime (1.16 g, 10 mmol) and 2-(piperidin-1-yl)ethanamine (1.47 g, 11.6 mmol) were mixed in 10 mL of ethanol. The reaction was stirred at 50 ºC overnight and cooled to room temperature. The precipitate was filtered under vacuum, washed with cold ethanol and rotary evaporated to a white solid. Yield: 63 %. ^{1}H NMR (400 MHz, DMSO-d_6) δ 11.92 (s, 1H), 7.92 (s, 1H), 8.07 (s, 2H), 3.24 (q, J = 8Hz, 2H), 2.37-2.32 (m, 6H), 1.51-1.45 (m, 4H), 1.40-1.35 (app d. J = 4, 2H).

S.2.3. RS69N encapsulation into MOF-808

In a 5 mL glass vial, 200 mg of **RS69N** (1 mmol) was dissolved in 0.4 mL 37 % HCl. The mixture was sonicated and rotary evaporated to an oil. Then, 20 mg **MOF-808** (0.01 mmol) and 1 mL of MeOH was added and stirred for 24 h. The white solid was washed with H_2O (2 x 1 mL) and recovered by centrifugation (9,168 g / 5 min). To remove the solvent, the material was lyophilized. In order to quantify the amount of **RS69N** encapsulated into **MOF-808**, ^{1}H spectra were recorded at room temperature. In a typical experiment, **RS69N@MOF-808** (20 mg) was mixed with 600 µL of deuterated NaOH 10 M and digested for a period of 24 h. The supernatant was collected by centrifugation (9,168 g / 5 min) and analysed by ^{1}H NMR spectra. TGA residue (calc./exp.): 30.8/35.8 % (**Figure S11**).

S.3. RS69N release from RS69N@MOF-808

20 mg of **RS69N@MOF-808** and 20 µL of dimethylacetamide (0.02 mmol), as internal reference, were suspended in 480 µL of deuterated PBS (100 mM). The concentration of released **RS69N** was followed by ^{1}H NMR spectra, at different times. In addition, the supernatant at 24 h was separated from the solid, mixed with 1 mL of deuterated NaOD 1 M and analysed by ^{1}H NMR, as well. All the experiments were performed by duplicate. **(Figure S4-S5, Table S1)**

S.4. Enzymatic assays

AChE activity was determined by using a colorimetric method based on the transformation of indoxyl acetate into indigo blue.[4] In a typical experiment 725 µL Tris-HCl (0.1 M, pH 7.5) and 25 µL of AChE aqueous solution (75 U/mL) were initially added in a 24 well culture plate. For the reactivation assays, 100 µL of an aqueous DIFP solution (final concentration $5\cdot10^{-6}$ M) and 100 µL of aqueous solution of free **RS69N** (final concentration $5\cdot10^{-3}$, $5\cdot10^{-4}$ or $5\cdot10^{-5}$ M) or 100 µL of the supernatant released from **RS69N@MOF-808** (final concentration $3.2\cdot10^{-3}$ M) were added to the well. Afterwards, 50 µL of indoxyl acetate solution in isopropanol were added as a substrate and the mixture was incubated for another 30 minutes. Finally, each sample was mixed with 3.33 mL of DMSO to fully solubilize the enzymatic product (indigo blue) and the enzymatic activity was determined spectrophotometrically (λ = 620 nm, $\epsilon_{indigo\ blue}$ = **22140** M^{-1} cm^{-1}) **(Table S2).**

The assays of inhibition enzymatic activity were carried out similarly than the reactivation assays. Specifically, a DIFP solution (0.028 M) and a suspension of RS69N@MOF-808 (20 mg) and DIFP (0.028 mM) in PBS were incubated at 37 ºC. After 24 hours of incubation, the supernatants were collected by centrifugation and diluted 560 times. Afterwards, 100 µL of the diluted-solutions (final DIFP concentration of $5\cdot10^{-6}$ M in negative control) were added to the well containing the enzyme solution (725 µL Tris-HCl (0.1 M, pH 7.5) and 25 µL of AChE aqueous solution (75 U/mL)) and the enzymatic activity was estimated following the indoxyl-acetate colorimetric method explained above **(Table S2).** All enzymatic assays were performed by triplicate.

S.5. DIFP removal studies

S.5.1. Gas chromatography studies

The adsorption of diisopropylfluorophosphate (DIFP) was studied by mixing **MOF-808** or **RS69N@MOF-808** (20 mg, 0.015 mmol), 2.5 µL of dimethyl acetamide (DMA, internal reference) and DIFP (2.5 µL, 0.015 mmol) in 500 µL of PBS (100 mM) in a closed vial with a septum. The evolution of the concentration of DIFP was followed at room temperature by means of Gas Chromatography taking 0.5 µL aliquots of the supernatant solution at each time.

The experimental data were fitted to a *pseudo-second* order model (Eq. 1):

$$q_t = \frac{{q_e}^2 \times k_t \times t}{1 + k_t \times t \times q_e} \quad (Eq. 1)$$

$t = time\ of\ adsorption\ (min)$

$q_t = adsorption\ capacity\ (mol \cdot mol^{-1})\ at\ time\ t$

$q_e = \ equilibrium\ adsorption\ capacity\ (mol \cdot mol^{-1})$

$k = adsorption\ rate\ constant\ (\ mol \cdot mol^{-1} \cdot min^{-1})$

S.5.2. ^{1}H and ^{31}P NMR studies

The adsorption/degradation of DIFP was also studied by ^{1}H and ^{31}P NMR. In a typical experiment, **MOF-808** or **RS69N@MOF-808** (20 mg, 0.015 mmol) and DIFP (2.5 µL, 0.015 mmol) were mixed in 500 µL of deuterated PBS. After 24 h, the supernatant was separated from the solid by centrifugation (2,486 *g* / 5 min) and the solid was suspended in DMSO-*d6* to extract the compounds trapped inside the cavities. Finally, both fractions (supernatant and extracted solution) were analysed by ^{1}H and ^{31}P NMR spectroscopy, using dimethylcetamide as an internal reference.

S.6. Computational modelling (Adsorbate locator)

Computational modelling of the interaction of **RS69N** oxime and DIFP with **MOF-808** host framework was performed with the BIOVIA Materials Studio 2018 Adsorption Locator Module (https://www.3ds.com/products-services/biovia/products/molecular-modeling-simulation/biovia-materials-studio/). Monte Carlo searches of the configurational space of the substrate-adsorbate system were carried out in order to

identify the most favourable adsorption configurations of the studied substrates in the MOF pore structure. We have explored oxime loadings of 1 to 52 oxime molecules per crystal cell which corresponds to 0.0625 to 3.25 oxime molecules per MOF formula unit. For DIFP molecule we have explored 0.0625 to 0.313 molecules per MOF formula unit.

FIGURES

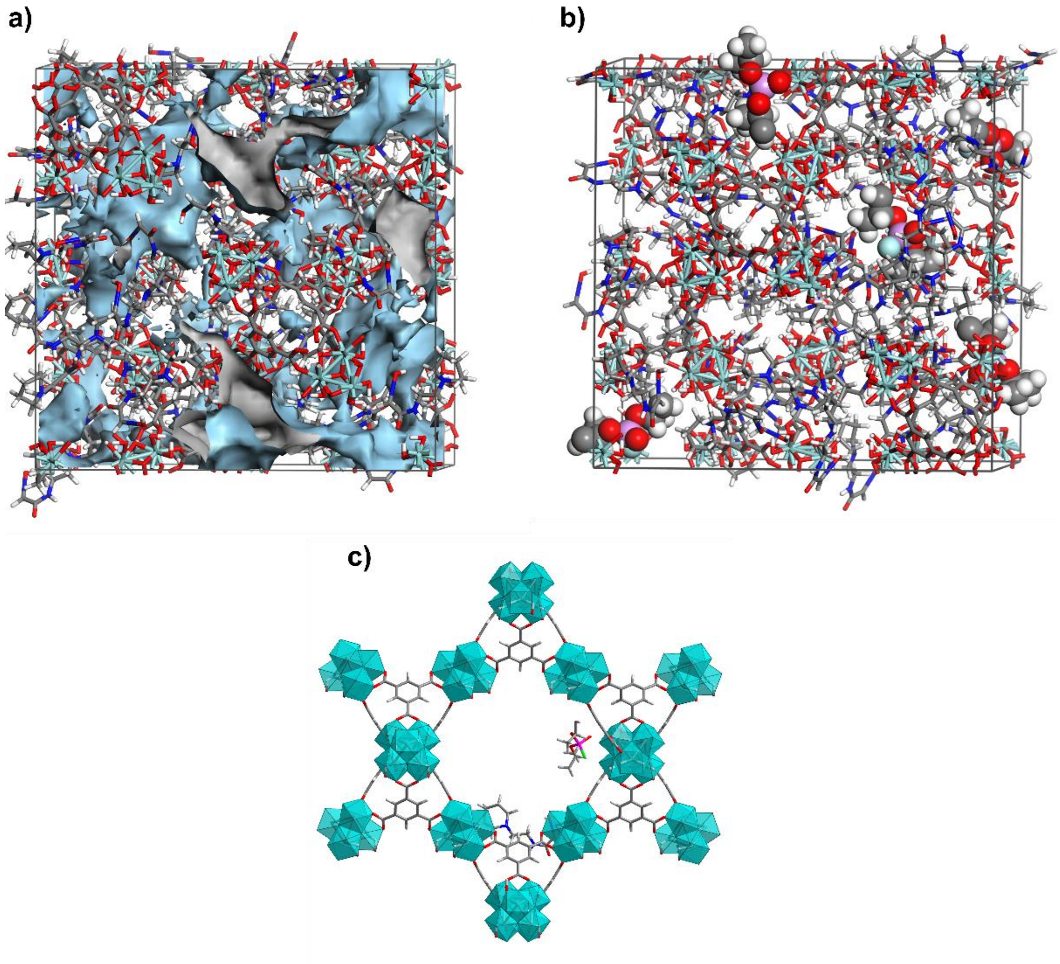

Figure S1. Computational model of: a) loading of 52 **RS69N** molecules per crystal cell highlighting the 7.36 % of accessible volume, b) loading of 52 **RS69N** molecules and 5 **DIFP** molecules per crystal cell and c) preferential co-adsorption of one **RS69N** molecule and one **DIFP** molecule in **MOF-808** porous structure.

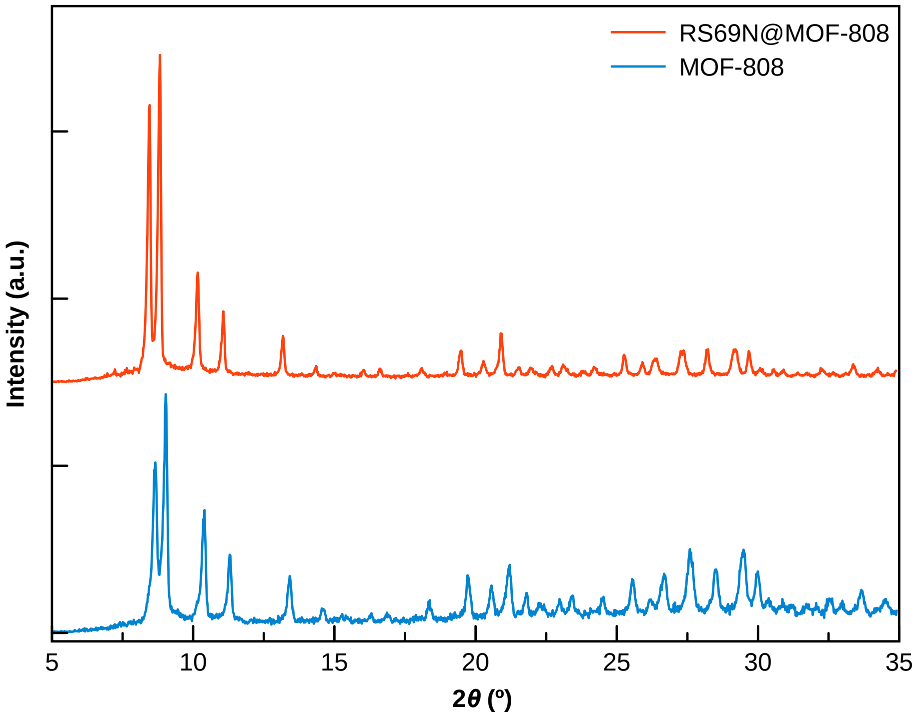

Figure S2. Powder X-ray diffractograms (PXRD) of **MOF-808** as synthesized (blue curve) and **RS69N@MOF-808** hybrid material (red curve).

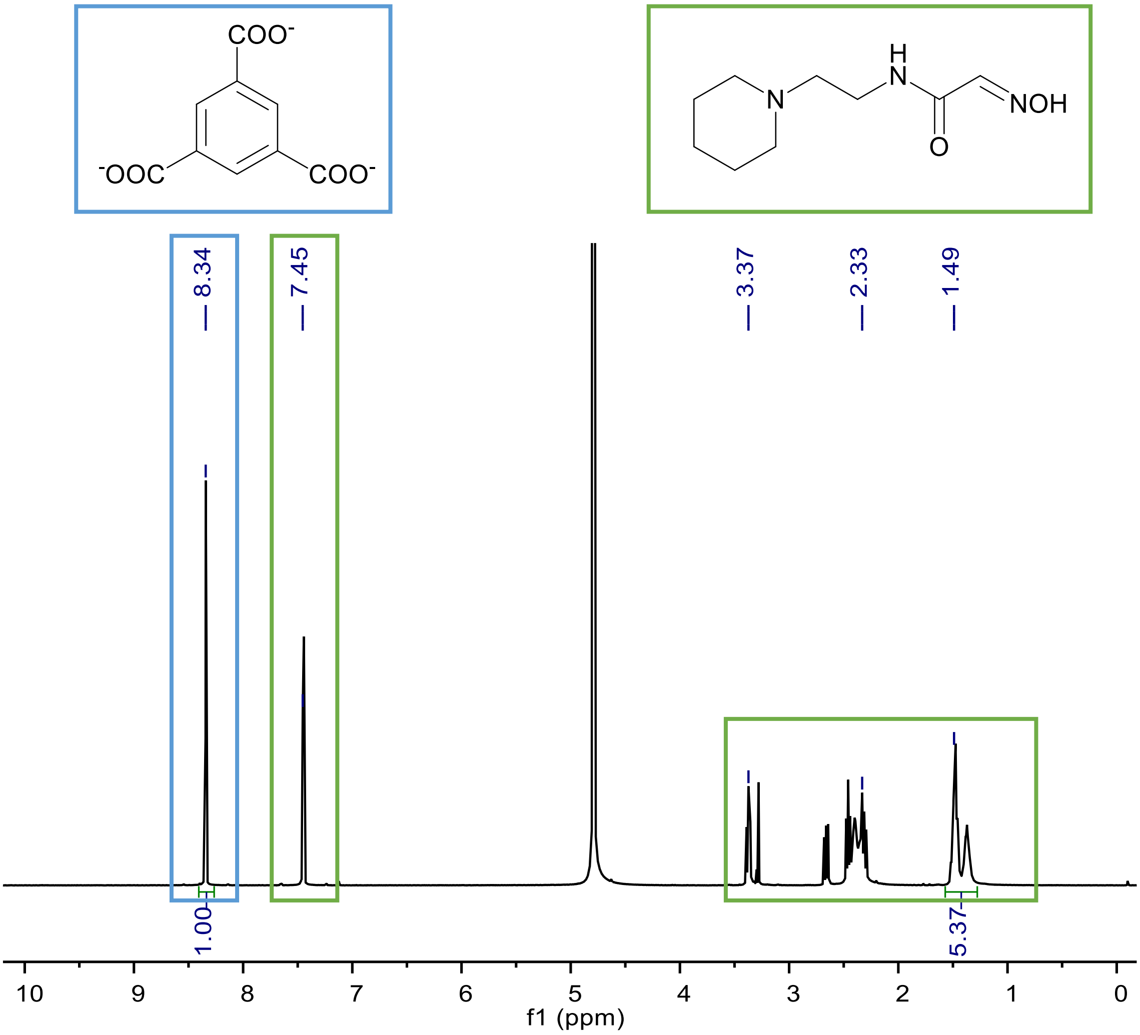

Figure S3. ^{1}H NMR spectra of the digestion of **RS69N@MOF-808** with NaOD solution. Experimental conditions: 20 mg of **RS69N@MOF-808**, 0.6 mL of NaOD (10 M), time of incubation = 24 h.

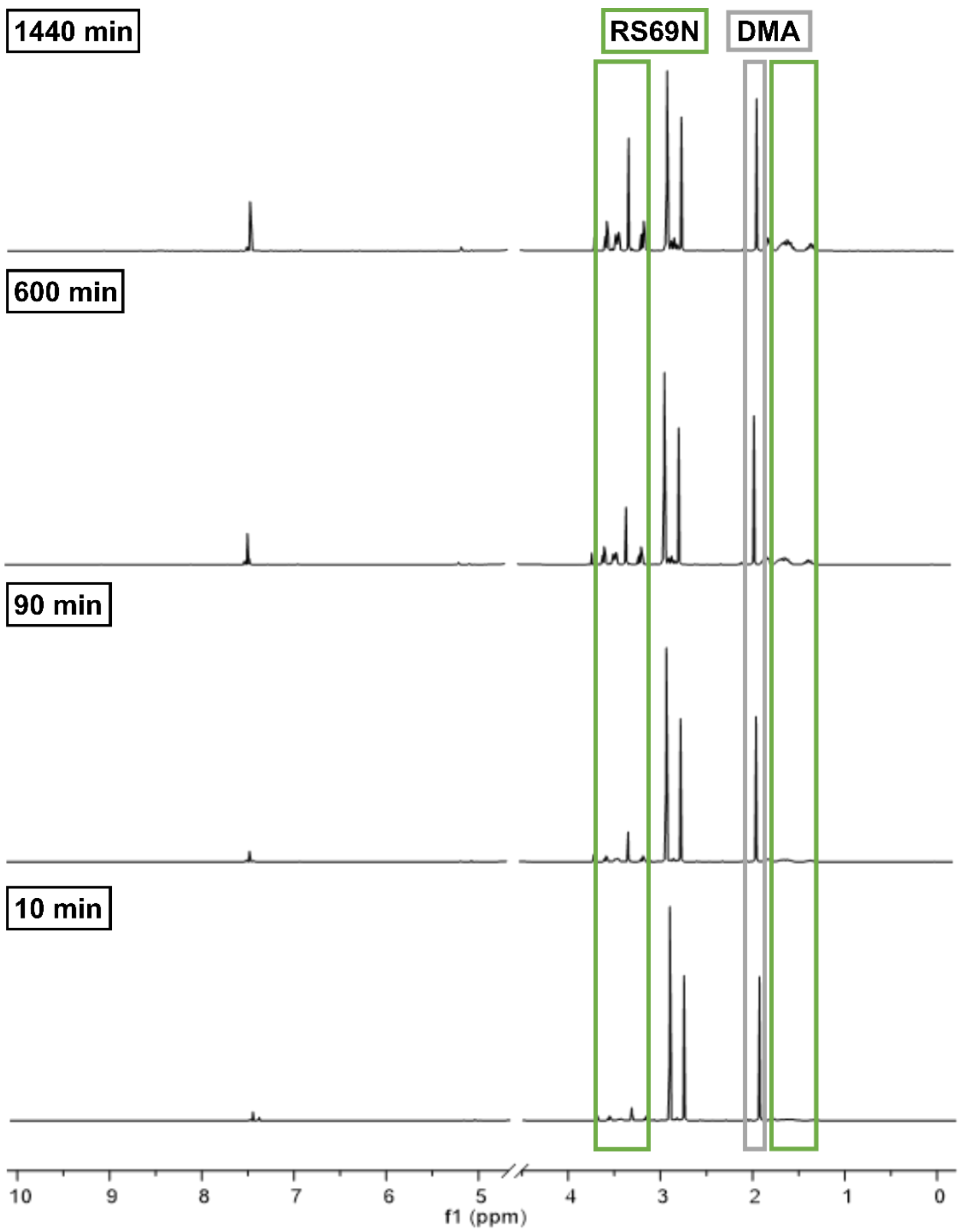

Figure S4. Oxime release from **RS69N@MOF-808:** ^{1}H NMR spectra after 10 min, 90 min, 600 min and 1440 min of incubation. Internal reference: N,N-Dimethylacetamide (DMA)

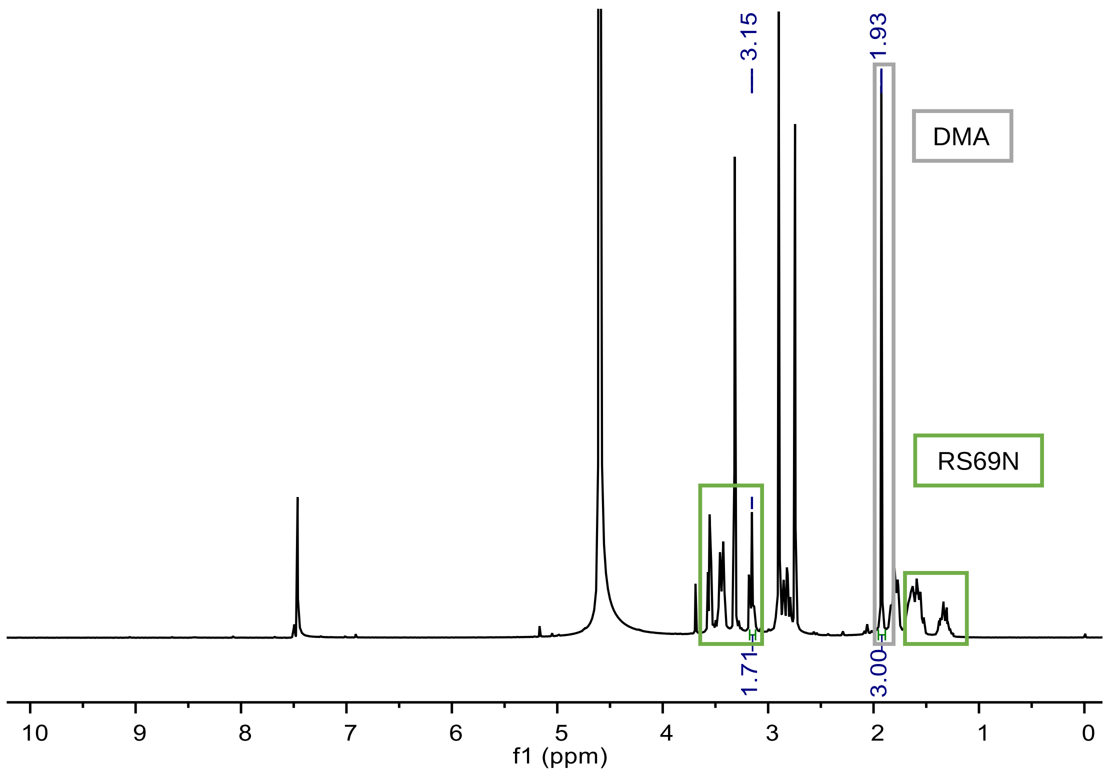

Figure S5. Oxime release from **RS69N@MOF-808:** ^{1}H NMR spectra after 24 h. NaOD solution (1 M) was added to fully deprotonate **RS69N** oxime.

Table S1. ^{1}H NMR integrated peak area corresponding to **RS69N** and internal reference (DMA) during oxime release from **RS69N@MOF-808** at different timeframes (10 min, 90 min, 600 min and 1440 min).

**Tiempo (min)**	**Integral DMA (δ1.93 ppm)**	**Integral RS69N (δ3.16 ppm)**
10 min	3.00	0.30
90 min	3.00	0.36
600 min	3.00	1.03
1440 min	3.00	1.61

Table S2. Enzymatic assays: Absorbances at λ=620 nm collected for the different enzymatic assays and calculate enzymatic activities. All experiments were performed by triplicate.

AChE reactivation of free RS69N at different concentrations

Sample	Absorbance (a.u.)	Enzymatic Activity (mU)
$Control_{enzyme}$	0.18 ± 0.01	1.07 ± 0.03
$Control_{inhibition}$	0.07 ± 0.01	0.41 ± 0.04
RS69N (5×10^{-3} M)	0.173 ± 0.003	1.04 ± 0.01
RS69N (5×10^{-4} M)	0.10 ± 0.01	0.56 ± 0.03

AchE reactivation of RS69N released from RS69N@MOF

Sample	Absorbance/min (a.u.)	Enzymatic Activity (mU))
$Control_{enzyme}$	0.277 ± 0.007	1.68 ± 0.04
$Control_{inhibition}$	0.088 ± 0.004	0.50 ± 0.02
RS69N@MOF-808	0.278 ± 0.001	1.69 ± 0.01

Enzymatic inhibition activities after detoxification with RS69N@MOF-808

Sample	Absorbance/min (a.u.)	Enzymatic Activity (mU))
$Control_{enzyme}$	0.179 ± 0.005	1.07 ± 0.03
$Control_{inhibition}$	0.075 ± 0.005	0.42 ± 0.03
RS69N@MOF-808	0.16 ± 0.01	0.93 ± 0.09

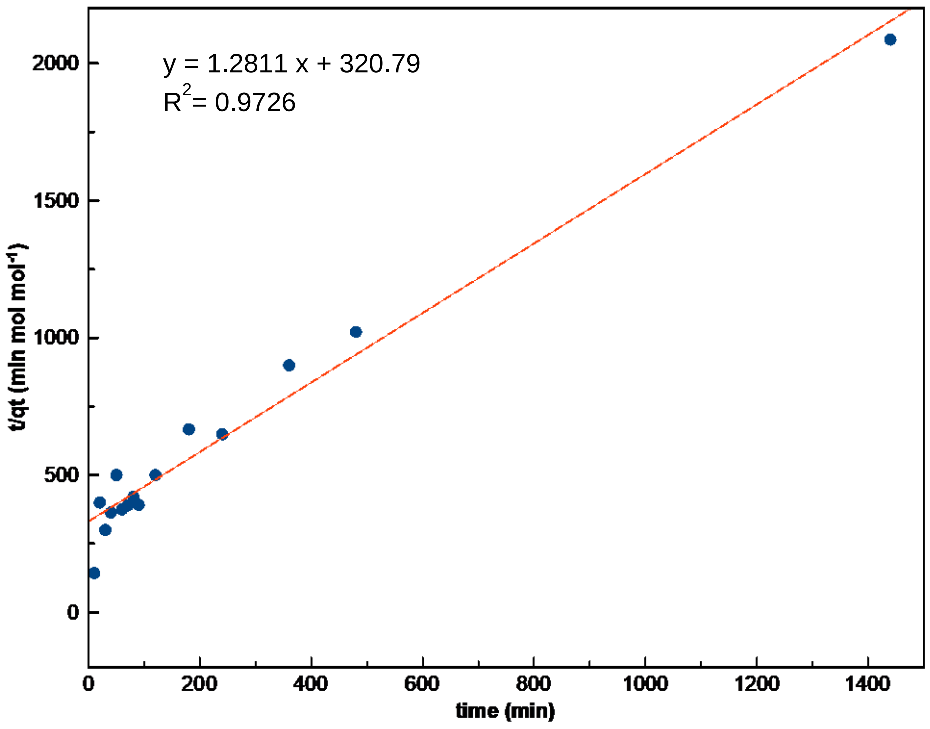

Figure S6. Fitting curves to pseudo-second order kinetics model for DIFP removal by **RS69N@MOF-808** hybrid material.

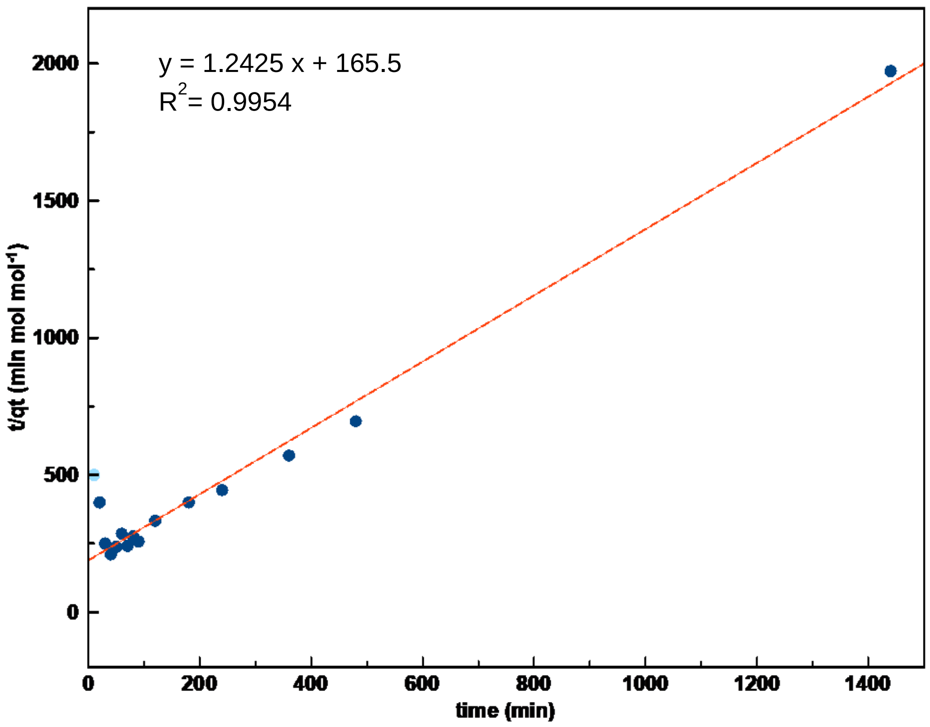

Figure S7. Fitting curves to pseudo-second order kinetics model for DIFP removal by **MOF-808**.

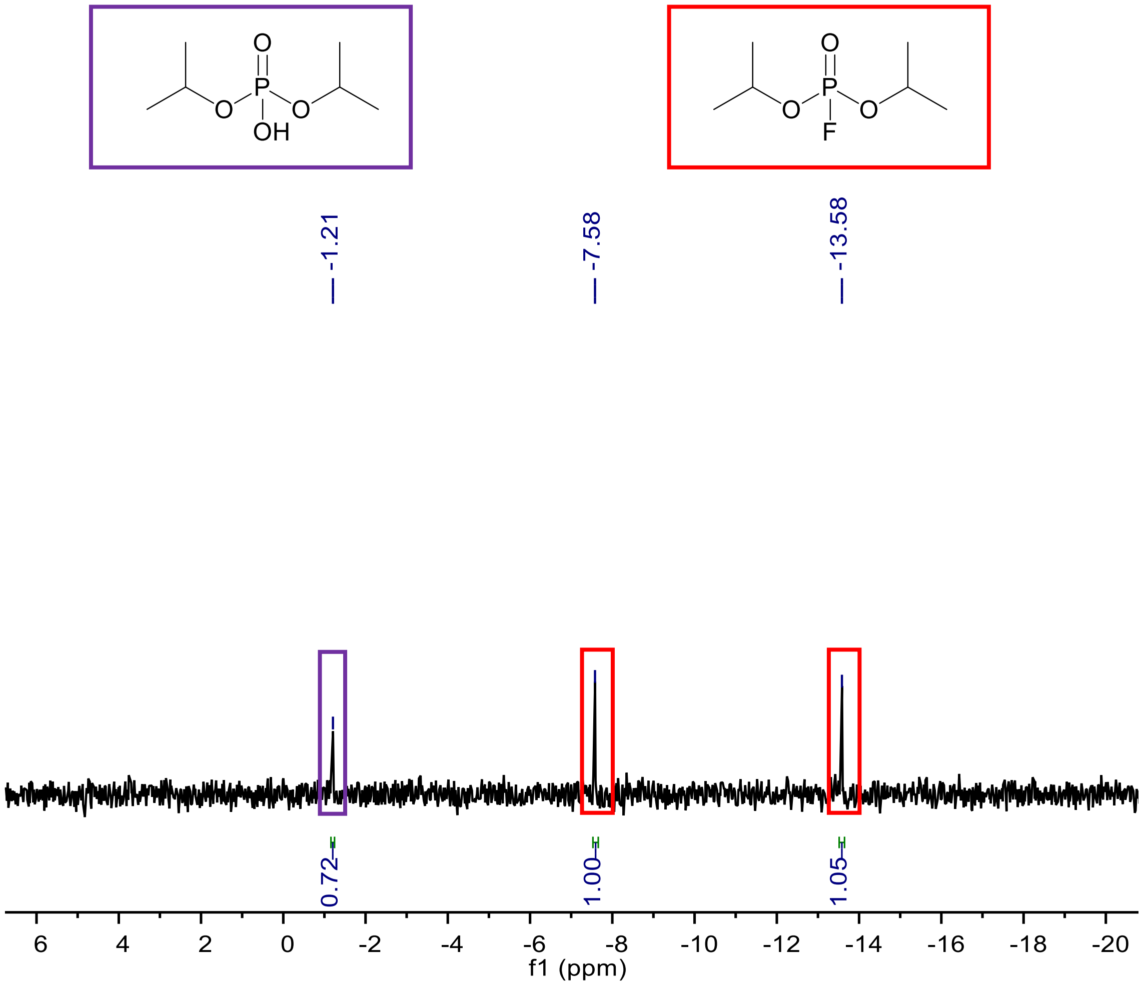

Figure S8. ^{31}P NMR spectra of supernatant after 24 h incubation of **RS69N@MOF-808** with DIFP in simulated physiological media. Experimental conditions: 20 mg of **RS69N@MOF-808**, [DIFP] = 0.028 M, 0.5 mL of deuterated PBS (100 mM, pH=7.4), room temperature.

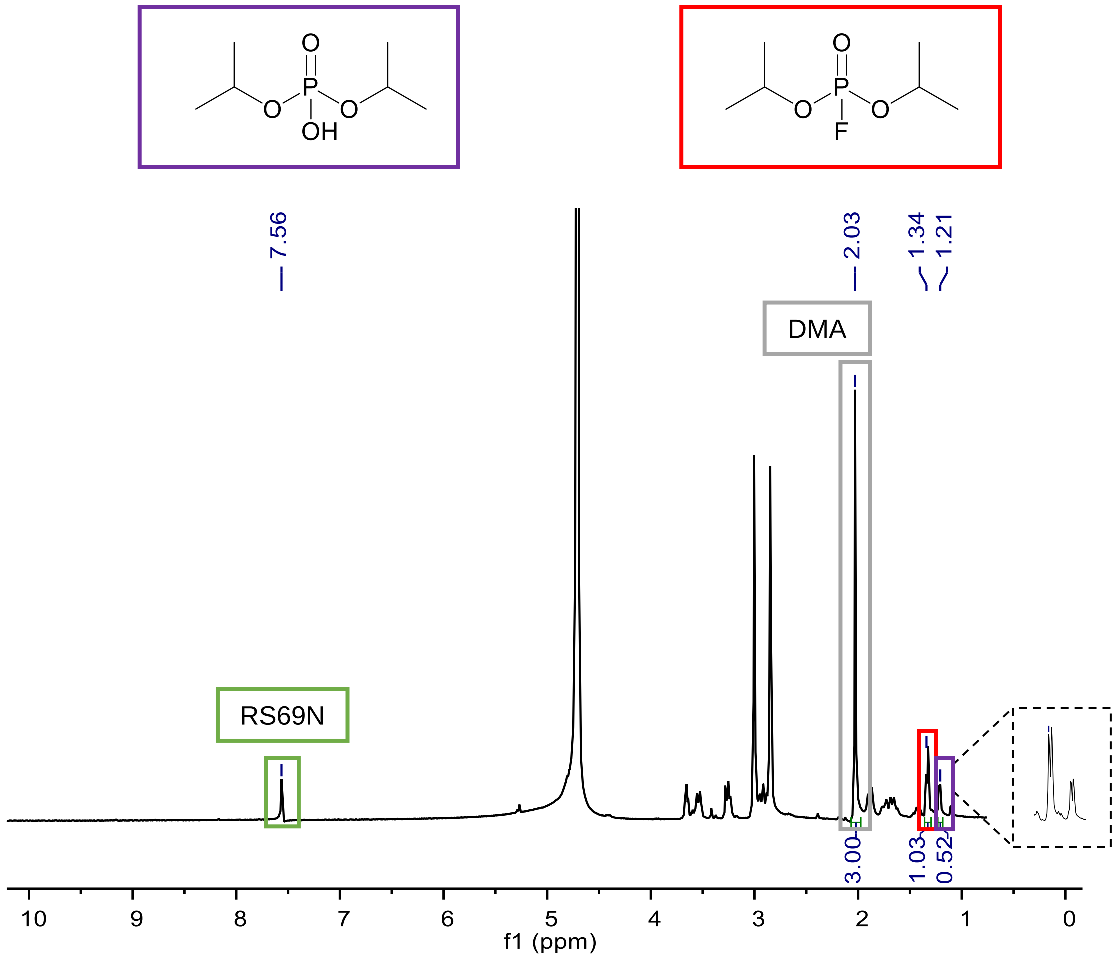

Figure S9. ^{1}H NMR spectra of supernatant after 24 h incubation of **RS69N@MOF-808** with DIFP in simulated physiological media. Experimental conditions: 20 mg of **RS69N@MOF-808**, [DIFP] = 0.028 M, 0.5 mL of deuterated PBS (100 mM, pH=7.4), room temperature.

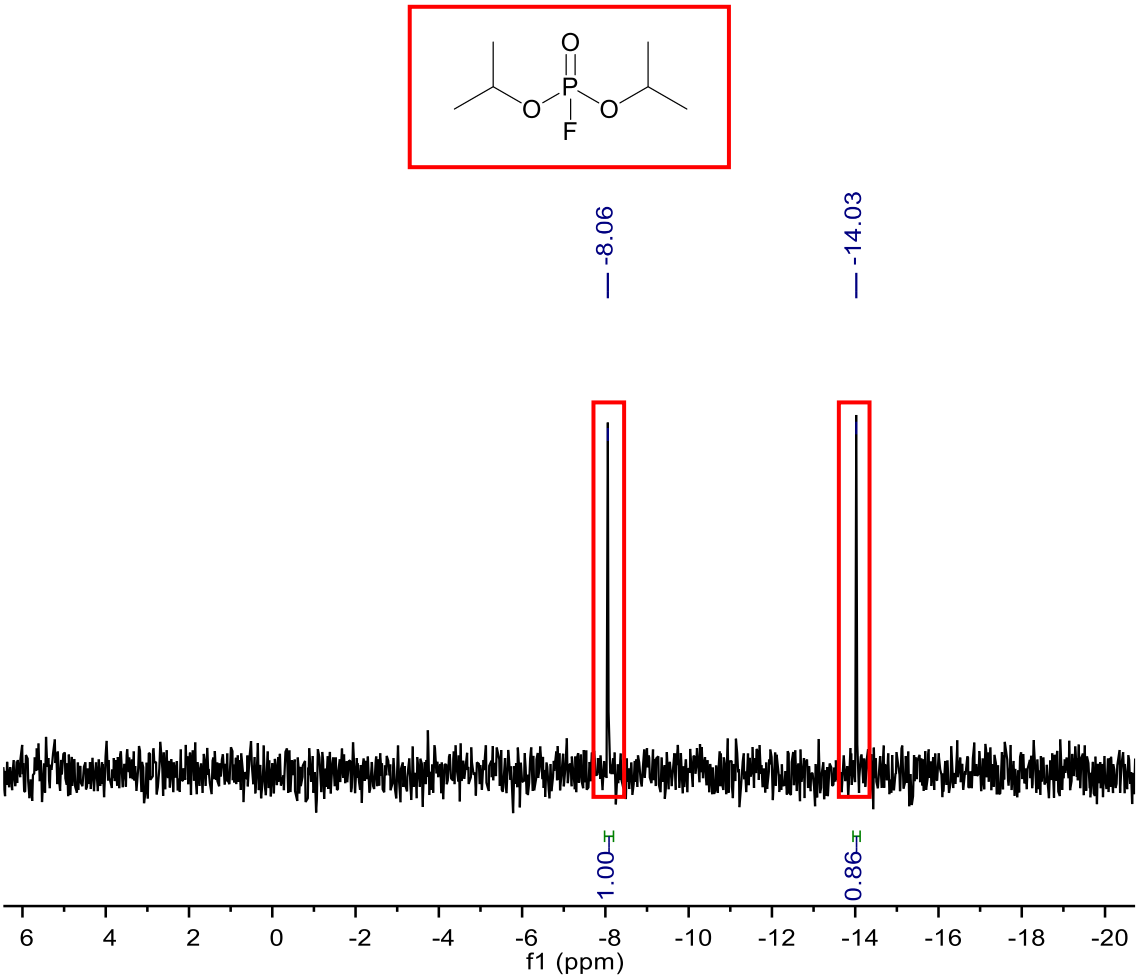

Figure S10. ^{31}P NMR spectra of extracted solution from **RS69N@MOF-808** solid previously incubated during 24 h with DIFP in physiological media. Extraction solvent: DMSO-d^6

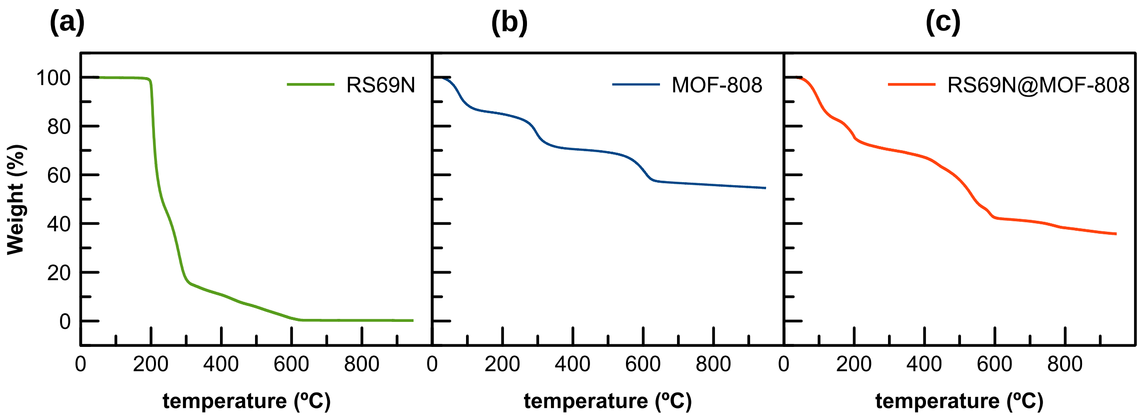

Figure S11. Thermogravimetric analysis of **RS69N** (green curve), **MOF-808** (blue curve) and **RS69N@MOF-808** (red curve).

BIBLIOGRAPHY

(1) González, L.; Gil-San-Millan, R.; Navarro, J. A. R.; Maldonado, C. R.; Barea, E.; Carmona, F. J. Green Synthesis of Zirconium MOF-808 for Simultaneous Phosphate Recovery and Organophosphorous Pesticide Detoxification in Wastewater. *J. Mater. Chem. A* **2022**. https://doi.org/10.1039/d2ta02074b.

(2) Mower, M. P.; Blackmond, D. G. Mechanistic Rationalization of Unusual Sigmoidal Kinetic Profiles in the Machetti-de Sarlo Cycloaddition Reaction. *J. Am. Chem. Soc.* **2015**, *137* (6), 2386–2391. https://doi.org/10.1021/ja512753v.

(3) Radić, Z.; Sit, R. K.; Kovarik, Z.; Berend, S.; Garcia, E.; Zhang, L.; Amitai, G.; Green, C.; Radić, B.; Fokin, V. V.; Sharpless, K. B.; Taylor, P. Refinement of Structural Leads for Centrally Acting Oxime Reactivators of Phosphylated Cholinesterases. *J. Biol. Chem.* **2012**, *287* (15), 11798–11809. https://doi.org/10.1074/jbc.M111.333732.

(4) Pohanka, M.; Hrabinova, M.; Kuca, K.; Simonato, J. P. Assessment of Acetylcholinesterase Activity Using Indoxylacetate and Comparison with the Standard Ellman’s Method. *Int. J. Mol. Sci.* **2011**, *12* (4), 2631–2640. https://doi.org/10.3390/ijms12042631.
